# Effectiveness of mRNA COVID-19 vaccines in preventing SARS-CoV-2 infections and COVID-19 hospitalisations and deaths in elderly long-term care facility residents, Spain, weeks 53 2020 to 13 2021

**DOI:** 10.2807/1560-7917.ES.2021.26.24.2100452

**Published:** 2021-06-17

**Authors:** Clara Mazagatos, Susana Monge, Carmen Olmedo, Lorena Vega, Pilar Gallego, Elisa Martín-Merino, María José Sierra, Aurora Limia, Amparo Larrauri, Working Group for the surveillance and control of COVID-19 in Spain, Working group for the surveillance and control of COVID-19 in Spain, Working group for the surveillance and control of COVID-19 in Spain, Luis Viloria, Alberto Malvar Pintos, Ana García-Fulgueiras, Ana Martínez Mateo, Ana Isabel Rivas Pérez, Nicola Lorusso, Araceli Alemán Herrera, Juan Pablo Alonso, Aurelio Barricarte, Magdalena Salom Castell, Cristina Ruiz Sopeña, Daniel Castrillejo, Eva Martínez Ochoa, Julián Mauro Ramos, Rosa Carbó Malonda, Ismael Huerta González, José Mª Arteagoitia Axpe, María Ordobás, Sara García Hernández.

**Affiliations:** 1 National Centre for Epidemiology, Institute of Health Carlos III, Madrid, Spain, Consortium for Biomedical Research in Epidemiology and Public Health (CIBERESP), Institute of Health Carlos III, Madrid, Spain; 2 Centre for the Coordination of Alerts and Health Emergencies, General Directorate of Public Health, Ministry of Health, Madrid, Spain; 3 Vaccines Division, General Directorate of Public Health, Ministry of Health, Madrid, Spain; 4 Spanish Agency of Medicines and Medical Devices-AEMPS, Madrid, Spain; 5 Members are listed under Investigators and at the end of the article

**Keywords:** COVID-19, vaccination, LTCF, surveillance, screening

## Abstract

Residents in long-term care facilities (LTCF) experienced a large morbidity and mortality during the COVID-19 pandemic in Spain and were prioritised for early COVID-19 vaccination. We used the screening method and population-based data sources to obtain estimates of mRNA COVID-19 vaccine effectiveness for elderly LTCF residents. The estimates were 71% (95% CI: 56–82%), 88% (95% CI: 75–95%), and 97% (95% CI: 92-99%), against SARS-CoV-2 infections (symptomatic and asymptomatic), and COVID-19 hospitalisations and deaths, respectively.

The coronavirus disease (COVID-19) pandemic has had a great impact on mortality in long-term care facilities (LTCF) in Spain. As of 4 April 2021, 30,176 COVID-19-related deaths have been reported among residents in these facilities [1]. When COVID-19 vaccination started on 27 December 2020, residents and LTCF personnel were prioritised for early vaccination with both mRNA COVID-19 vaccines, Comirnaty (BNT162b2, BioNTech/Pfizer, Mainz, Germany/New York, United States (US)) and Moderna (mRNA-1273, Moderna, Cambridge, US). As of 4 April 2021, 300,133 (88.8%) elderly residents (aged 65 years and older) in LTCF had received the second dose and were fully vaccinated, according to the National COVID-19 Vaccination Registry (REGVACU) [2].

The screening method provides a simple and rapid surveillance tool for monitoring the effectiveness of vaccines over time [3,4]. This method is particularly useful in the pandemic context to obtain early estimates of mRNA COVID-19 vaccine effectiveness (VE) as it utilises data already available on SARS-CoV-2 infections and doses of COVID-19 vaccines administered in the population. We estimated the effectiveness of vaccination in preventing symptomatic and asymptomatic SARS-CoV-2 infections, as well as COVID-19 hospitalisations and deaths in elderly LTCF residents in Spain, using the screening method and population-based data sources.

## Case definition and proportion of COVID-19 vaccinated elderly long-term care facility residents

**Figure 1:**
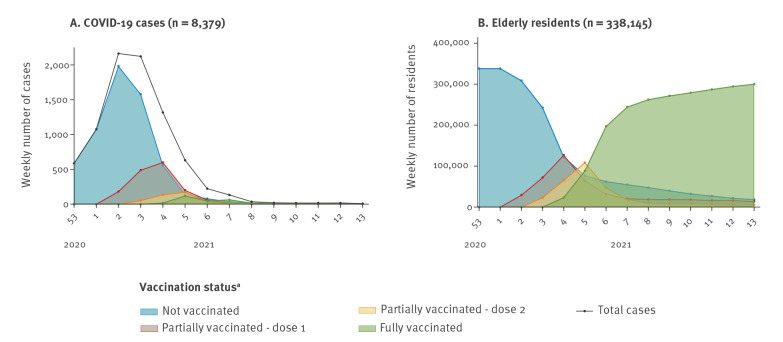
Weekly number of (A) COVID-19 cases and (B) elderly long-term care facilities residents by COVID-19 vaccination status, Spain, weeks 53 2020 to 13 2021

We obtained the weekly number of cases in elderly LTCF residents from the National Epidemiological Surveillance Network (RENAVE) by vaccination status, from 27 December 2020 to 4 April 2021 (weeks 53 2020–13 2021) (Figure 1A). As LTCF data are not integrated in RENAVE, cases in elderly residents of LTCF were defined as: (i) aged 65 years and older, (ii) COVID-19 exposure in an institutional or residential setting, and (iii) not being a social or healthcare worker. We discarded data from autonomous regions with no information on age, exposure setting, healthcare worker status or COVID-19 vaccination status. We included data from 12 of 19 autonomous regions in the analysis, representing 66.1% of the total Spanish population. Residents with SARS-CoV-2 infections (symptomatic or asymptomatic) (n = 8,379), including asymptomatic infections (n = 3,470), hospitalisations (n = 2,509), and deaths (n = 1,602) with a positive COVID-19 test were included. Among the SARS-CoV-2 infections, 82% were PCR-confirmed and 18% were rapid antigen test-confirmed. These n numbers were used as the denominators to calculate the proportion of cases vaccinated (PCV) and, to obtain the numerators, individuals were classified into four mutually exclusive categories of vaccination status (Box).

COVID-19: Coronavirus disease.

^a^ Definitions of COVID-19 vaccination status are in the Box.

Box **Definitions of COVID-19 vaccination status in elderly long-term care facility residents**

**Not vaccinated:** Not vaccinated with any dose of Comirnaty (BNT162b2 mRNA, BioNTech-Pfizer)^a^ or Moderna COVID-19 vaccine (mRNA-1273)^b^ or 14 days or less since vaccination with the first dose.

**Partially vaccinated - dose 1:** Vaccinated with the first dose of Comirnaty or Moderna COVID-19 vaccine, and more than 14 days since vaccination.

**Partially vaccinated - dose 2:** Vaccinated with two doses of Comirnaty or Moderna COVID-19 vaccine, and less than 7 days since the second dose for Comirnaty or less than 14 days for Moderna COVID-19 vaccine. Full immunity not reached.

**Fully vaccinated:** Vaccinated with two doses, and 7 days or more after the second dose for Comirnaty and 14 days or more for Moderna COVID-19 vaccine. Full immunity reached.

COVID-19: coronavirus disease.
^a^ BioNTech-Pfizer, Mainz, Germany/New York, United States (US)
^b^ Moderna, Cambridge, US

## Proportion of vaccinated elderly long-term care facility residents

REGVACU is a nationwide registry of all COVID-19 vaccine doses administered and rejected. All records of people aged 65 years and older living in LTCF and vaccinated between 27 December 2020 and 4 April 2021 were included and categorised by vaccination status (Figure 1B). The population of LTCF residents aged 65 years and older in Spain was the denominator for the weekly proportion of the population vaccinated (PPV). We estimated this figure from the overall institutionalised population (any age and type of institution) [2] as follows: using REGVACU, we calculated that, of all institutionalised people who received the first dose, 82.7% were aged 65 years and older. Then, we applied this percentage to the overall institutionalised population to obtain the number of residents in LTCF aged 65 years and older by region, as denominators for PPV. We estimated a total of 338,145 residents in LTCF aged 65 years and older in Spain.

## Vaccine effectiveness in elderly long-term care facility residents

We present the national weekly number of fully vaccinated COVID-19 cases in elderly LTCF residents, as well as the weekly proportion of cases vaccinated (PCV) and PPV for each disease outcome (Table 1).

**Table 1:** Weekly number and proportion of SARS-CoV-2-associated infections, hospitalisations and deaths among fully vaccinated^a^ elderly long-term care facility residents and weekly proportion of fully vaccinated^a^ elderly long-term care facility residents, Spain, weeks 53 2020 to 13 2021

Weeks	Outcomes in fully vaccinated^a^ LTCF residents								Fully vaccinated^a^ LTCF population (n = 338,145)	
	SARS-CoV-2 infections^b^		Asymptomatic SARS-CoV-2 infections		Hospitalisations		Deaths			
	n	PCV (%)	n	PCV (%)	N	PCV (%)	n	PCV (%)	n	PPV (%)
53/2020	0	0.0	0	0.0	0	0.0	0	0.0	0	0.0
1/2021	0	0.0	0	0.0	0	0.0	0	0.0	0	0.0
2/2021	0	0.0	0	0.0	0	0.0	0	0.0	0	0.0
3/2021	0	0.0	0	0.0	0	0.0	0	0.0	0	0.0
4/2021	16	1.2	4	0.6	4	1.3	4	1.1	23,204	6.9
5/2021	118	18.7	70	23.8	12	7.9	12	4.4	88,557	26.2
6/2021	47	21.2	35	36.5	7	10.1	7	10.0	197,104	58.3
7/2021	63	47.7	48	67.6	8	25.0	8	27.3	244,270	72.2
8/2021	16	45.7	7	58.3	2	16.7	2	0.0	262,363	77.6
9/2021	11	57.9	6	100.0	4	40.0	4	25.0	271,620	80.3
10/2021	7	43.8	3	42.9	3	42.9	3	100.0	279,220	82.6
11/2021	6	37.5	2	50.0	4	44.4	4	66.7	287,219	84.9
12/2021	4	23.5	0	0.0	3	37.5	3	100.0	294,583	87.1
13/2021	4	50.0	2	50.0	2	66.7	2	0.0	300,133	88.8

COVID-19: coronavirus disease; LTCF: long-term care facilities; PCV: proportion of cases vaccinated; PPV: proportion of population vaccinated; SARS-CoV-2: severe acute respiratory syndrome coronavirus 2.

^a^ Definitions of COVID-19 vaccination status are in the Box.

^b^ Symptomatic and asymptomatic SARS-CoV-2 infections.

The VE and 95% confidence interval (CI) were calculated using the screening method, according to Farrington [3]. We estimated the odds ratio (OR) of vaccination for cases compared with population, as previously described [5]. Using the following formula, VE was calculated as 1 − OR:

$$VE=1-\left(\frac{PCV}{\left(1-PCV\right)}\times \frac{\left(1-PPV\right)}{PPV}\right)$$

**Figure 2:**
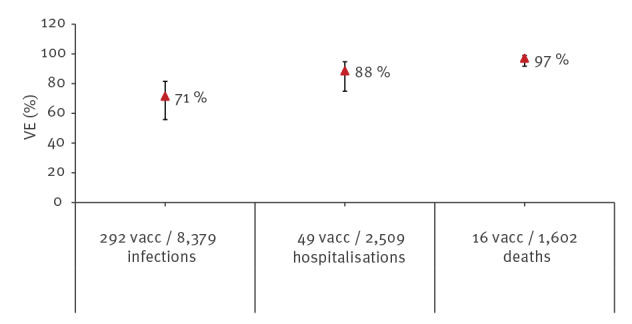
Vaccine effectiveness against SARS-CoV-2 infections, hospitalisations and deaths in fully vaccinated^a^ elderly long-term care facility residents, Spain, weeks 53 2020 to 13 2021 (n = 8,379)

COVID-19: coronavirus disease; Vacc: fully vaccinated; VE: vaccine effectiveness.

^a^ Definitions of COVID-19 vaccination status are in the Box.

We assumed a time lag of a full vaccination course to have an impact in the prevention of hospitalisations and deaths. Thus, for the outcomes studied, we compared the PPV of each week (n) with the PCV in COVID-19 hospitalisations and deaths corresponding to 1 (n + 1) and 2 weeks later (n + 2), respectively. The VE in preventing symptomatic or asymptomatic SARS-CoV-2 infections was 50.5% (95% CI: 37.1%–61.1%), 78.7% (95% CI: 67.0%–86.2%) and 71.4% (95% CI: 55.7%–81.5%), among those partially vaccinated with dose 1, partially vaccinated with dose 2, and fully vaccinated, respectively. All estimates were similar when restricting analyses to asymptomatic SARS-CoV-2 infections (Table 2). In fully vaccinated LTCF residents, VE was 88.4% (95% CI: 74.9%–94.7%) and 97.0% (95% CI: 91.7%–98.9%) in preventing COVID-19 hospitalisations and deaths, respectively (Table 2 and Figure 2).

**Table 2:** Number of vaccinated and total COVID-19 cases, proportion of cases vaccinated and COVID-19 vaccine effectiveness in preventing symptomatic and asymptomatic SARS-CoV-2 infections, and COVID-19 hospitalisations and deaths in elderly long-term care facility residents by vaccination status^a^, Spain, weeks 53 2020 to 13 2021 (n = 8,379)

Disease outcomes	Vaccination status^a^	Vaccinated/ total cases	PCV (%)	VE (95% CI)
SARS-CoV-2 infections^b^	Partially vaccinated - dose 1	1,559/8,379	18.6	50.5% (37.1%–61.1%)
	Partially vaccinated - dose 2	413/8,379	4.9	78.7% (67.0%–86.2%)
	Fully vaccinated	292/8,379	3.5	71.4% (55.7%–81.5%)
Asymptomatic SARS-CoV-2 infections	Partially vaccinated - dose 1	634/3,470	18.3	58.0% (41.7%–69.7%)
	Partially vaccinated - dose 2	178/3,470	5.1	84.7% (71.9%–91.7%)
	Fully vaccinated	177/3,470	5.1	69.7% (47.7%–82.5%)
Hospitalisations^b^	Partially vaccinated - dose 1	404/2,509	16.1	53.0% (25.7%–70.3%)
	Partially vaccinated - dose 2	101/2,509	4.0	83.0% (61.2%–92.6%)
	Fully vaccinated	49/2,509	2.0	88.4% (74.9%–94.7%)
Deaths^b^	Partially vaccinated - dose 1	236/1,602	14.7	55.6% (26.6%–73.2%)
	Partially vaccinated - dose 2	35/1,602	2.2	95.7% (82.6%–98.9%)
	Fully vaccinated	16/1,602	1.0	97.0% (91.7%–98.9%)

CI: confidence interval; COVID-19: coronavirus disease; PCV: proportion of cases vaccinated; VE: vaccine effectiveness; SARS-CoV-2: severe acute respiratory syndrome coronavirus 2.

^a^ Definitions of COVID-19 vaccination status are in the Box.

^b^ Symptomatic and asymptomatic SARS-CoV-2 infections. Hospitalisations and deaths are among COVID-19 cases.

All estimates are adjusted by autonomous region.

Fully vaccinated symptomatic and asymptomatic SARS-CoV-2 ‘infections’, and COVID-19 ‘hospitalisations’ and ‘deaths’ refer to total number of COVID-19 cases with each outcome. All estimates are adjusted by autonomous region.

## Discussion

COVID-19 mRNA vaccines Comirnaty and Moderna, administered in the first phase of the vaccine rollout in Spain, were highly effective in preventing not only SARS-CoV-2 infections, but also hospitalisations and deaths in elderly LTCF residents. Using the screening approach, we estimated a considerable VE (50.5%) with the first dose of Comirnaty and Moderna against SARS-CoV-2 infections, as was also observed (56% to 62%) in a cohort study in the UK [6], and VE increased in those fully vaccinated. Notably, the protection against SARS-CoV-2 asymptomatic infections was similar to that for symptomatic infections, which is consistent with previously reported data on mRNA-based vaccines [7]. Given the role of asymptomatic infection on transmission [8], a high VE against asymptomatic infection may serve as indirect evidence of the contribution of vaccines in reducing viral spread in the community.

Our VE estimates for COVID-19 hospitalisations and deaths support evidence from other countries favouring the use of mRNA COVID-19 vaccines to prevent severe disease outcomes. Two studies from a nationwide mass vaccination setting in Israel have shown high effectiveness of the Comirnaty vaccine against a range of COVID-19 outcomes [9,10]. Also, a multicentre test-negative case–control study in a population aged 65 years and older hospitalised across the US showed a 64% and 94% VE of mRNA vaccines in preventing hospitalisations following the first and second dose, respectively [11].

Most observational studies estimating COVID-19 VE have used test-negative or cohort designs [9,11]. Nevertheless, when timely surveillance data are available and vaccine coverage in the population is robustly collected, as in Spain with REGVACU, the screening approach is an appropriate and readily available methodology for early VE estimation against different disease outcomes [3-5]. We find that our nationwide results of VE using this method (71.4% against SARS-CoV-2 infection, 88.4% and 97.0% against COVID-19 hospitalisations and deaths, respectively) are in the range of previous analyses (81%) in Spain, which used more robust cohort study designs in elderly LTCF residents and focused on direct and indirect effects of mRNA vaccines against SARS-CoV-2 infections [12]. Also, our results are in accordance with other estimates at regional level that examined the prevention of hospitalisations and deaths (95% and 97%, respectively) in elderly LTCF residents and healthcare workers [13] or against symptomatic infections in a population aged 60 years and older (77%) [14].

However, using COVID-19 surveillance information also has limitations because of data reporting and quality issues. Information on cases in LTCF is not integrated in the national COVID-19 surveillance, and we based our case definitions on several variables collected within the RENAVE. Therefore, regions with better compliance in reporting key study variables to the national surveillance may be overrepresented among our cases. Also, regional vaccination registries may have specific reporting issues. To control for this variability between regions, we adjusted the analysis by autonomous region, whenever the sample size allowed. Also, we did not include data in the analyses from autonomous regions lacking information on variables defining elderly LTCF residents.

Another major shortcoming of the screening method is that data from cases and the control group come from different sources. However, in this study, both data sources –RENAVE for cases and REGVACU for vaccine coverage – are comprehensive registries of national coverage based on compulsory data reporting from all autonomous regions, allowing for a national VE estimation.

In conclusion, COVID-19 vaccination using mRNA vaccines in Spain was very effective in preventing SARS-CoV-2 infections, and COVID-19 hospitalisations and deaths in elderly LTCF residents. The similar level of protection against asymptomatic and symptomatic infections among fully vaccinated LTCF residents may serve as indirect evidence of the contribution of vaccines in reducing viral spread in the community. As the vaccination campaign continues, additional studies will be necessary in order to address the effects of COVID-19 vaccination against emerging SARS-CoV-2 variants and in other population groups and to inform public health response.

## Working group for the surveillance and control of COVID-19 in Spain

Luis Viloria (Sección de Epidemiología, Consejería de Sanidad-Gobierno de Cantabria, Santander)

Alberto Malvar Pintos (Servicio de Epidemiología, Dirección Xeral de Saúde Pública, Santiago de Compostela, Galicia)

Ana García-Fulgueiras (Servicio de Epidemiología, Dirección General de Salud Pública y Adicciones, Murcia)

Ana Martínez Mateo (Subdirección General de Vigilancia y Respuesta a Emergencias de Salud Pública, Agencia de Salud Pública, Catalunya and CIBER Epidemiologia y Salud Pública (CIBERESP))

Ana Isabel Rivas Pérez (Servicio de Epidemiología, Consejería de Sanidad, Consumo y Gobernación, Ceuta)

Nicola Lorusso (Dirección General de Salud Pública y Ordenación Farmacéutica, Sevilla, Andalucía)

Araceli Alemán Herrera (Dirección General de Salud Pública, Islas Canarias)

Juan Pablo Alonso (Servicio de Vigilancia en Salud Pública, Dirección General de Salud Pública, Zaragoza, Aragón)

Aurelio Barricarte (Sección de Vigilancia de Enfermedades Transmisibles del Instituto de Salud Pública, CIBERESP, Pamplona, Navarra)

Magdalena Salom Castell (Direcció General Salut Pública i Participació, Illes Balears)

Cristina Ruiz Sopeña (Servicio de Epidemiología, Dirección General de Salud Pública, Castilla y León)

Daniel Castrillejo (Servicio de Epidemiología, Dirección General de Salud Pública y Consumo, Melilla)

Eva Martínez Ochoa (Servicio de Epidemiología y Prevención Sanitaria, Dirección General de Salud Pública, Consumo y Cuidados, La Rioja)

Julián Mauro Ramos (Dirección General de Salud Pública, Extremadura)

Rosa Carbó Malonda (Servicio de Vigilancia y Control Epidemiológico, Dirección General de Salud Pública y Adicciones, Comunidad Valenciana)

Ismael Huerta González (Servicio de Vigilancia Epidemiológica, Dirección General de Salud Pública, Asturias)

José Mª Arteagoitia Axpe (Servicio de Vigilancia y Vacunas, Dirección de Salud Pública y Adicciones, País Vasco)

María Ordobás (Subdirección General de Epidemiología, Dirección General de Salud Pública, Comunidad de Madrid)

Sara García Hernández (Servicio Epidemiología, Castilla-La Mancha)
